# Short, stringent lockdowns halted SARS-CoV-2 transmissions in Danish municipalities

**DOI:** 10.1038/s41598-024-68929-z

**Published:** 2024-08-12

**Authors:** Florian Ege

**Affiliations:** Interdisciplinary Centre on Population Dynamics, University of Southern Denmark, Odense, Denmark

**Keywords:** Infectious diseases, Epidemiology, Outcomes research

## Abstract

In late 2020, the focus of the global effort against the COVID-19 pandemic centered around the development of a vaccine, when reports of a mutated SARS-CoV-2 virus variant in a population of 17 million farmed mink came from Denmark, threatening to jeopardize this effort. Spillover infections of the new variant between mink and humans were feared to threaten the efficacy of upcoming vaccines. In this study the ensuing short-lived yet stringent lockdowns imposed in 7 of the countries 98 municipalities are analysed for their effectiveness to reduce SARS-CoV-2 infections. Synthetic counterfactuals are created for each of these municipalities using a weighted average combination of the remaining municipalities not targeted by the stringent measures. This allows for a clear overview regarding the development of test-positivity rates, citizen mobility behaviours and lastly daily infection numbers in response to the restrictions. The findings show that these targeted, short-term lockdowns significantly curtailed further infections, demonstrating a marked decrease, first in citizens mobility and then in daily cases when compared to their synthetic counterfactuals. Overall, the estimates indicate average reductions to infection numbers to be around 31%. This study underscores the potential of strict, yet severe lockdowns in breaking ongoing infection dynamics, by utilising a rare quasi-experimental design case that avoids bias introduced through treatment selection.

## Introduction

Estimating the effectiveness of COVID-19 non-pharmaceutical interventions (NPIs) employed to reduce infections, hospitalizations and ultimately death, presents a substantial challenge^1^. Given that randomized controlled trials, the gold standard for evaluating policy effectiveness, are not feasible, studies adopting quasi-experimental designs emerge as viable alternatives^2^. However, the substantial direct and indirect effects of NPIs, for example on mental health^3^ or the economy^4^, mean such measures are implemented only when strictly necessary. This in turn means that policymakers target NPIs at geographical areas that exhibit high infection numbers and adjust their intensity based on the given severity. This selective deployment introduces an endogeneity bias. Here, for example a region exhibiting elevated levels of SARS-CoV-2 infections will be targeted by more stringent NPIs. However, this targeted process renders these cases unsuitable for quasi experimental analysis as the selection criteria (elevated prior infection levels) is directly correlated with the outcome measure (infection levels)^5^. This means that unobserved underlying causes The following study presents a rare case in which policymakers implemented a stringent lockdown, not due to high infection numbers but out of concern about a novel virus-variant that was feared to potentially undermine future vaccination efforts. Thereby, the case avoids said endogeneity bias and can serve as a quasi-experiment to investigate the actual impact of a lockdown on SARS-CoV-2 infection numbers.

Previous studies have managed to find quasi-experimental designs to estimate the effectiveness of specific restrictions. Mitze et al. were able to isolate the effect of an early implementation of face mask mandates in a German region, finding about a 45% reduction in SARS-CoV-2 infections^6^. A study of lagged school re-openings in Italian regions was able to find a positive effect of earlier re-openings on local infection numbers^7^. Another study, utilising an administrative error in English contact tracing data, was able to estimate the effect of contact tracing to be about a 63% reduction in infection numbers^8^. For estimating the effect of a lockdown policy itself, some studies have used the case of the capital of Chile, Santiago^9,10^. Here policymakers decided to implement lockdowns in parts of the city that showcased particularly high infection numbers, raising concern about the exogeneity of the cases treatment selection process.

This study adds to the literature, by estimating the effects of a lockdown in a quasi experimental case in Denmark. At the end of 2020, Denmark was one of the clear global leaders in SARS-CoV-2 testing, making its infection numbers among the most credible^11,12^. Given the first roll-outs of COVID-19 vaccines in the end of 2020, its timing is ideal. As the lockdown occurred later into the first year of the pandemic, testing capacities were much more developed. The effects of the lockdown can be estimated right before the first vaccines were rolled out in Denmark, making its findings independent of any potential effects vaccinations might have on the estimation of the effectiveness of lockdowns.

Two previous studies have investigated the specific case. A study by Dall Schmidt & Mitze focused on the epidemiological link between animal-to-human and human-to-human viral transmission finding a substantial effect of culling the local mink population, from which the virus-variant of concern originated, when done in combination with a lockdown^13^. The second study investigated the lockdowns effect on SARS-CoV-2 transmissions and found these to be limited and statistically insignificant when compared to neighbouring municipalities^14^.

On November 2nd, 2020, the Danish National Epidemiological Institute (Statens Serum Institute (SSI)) issued a report expressing concern about a mutated SARS-CoV-2 variant that had been detected in the country’s extensive population of farmed mink and from there had spread to its human population^15^. At this point in time, vaccines were expected ready by early 2021 and therefore, reports about the mutation’s potential to reduce vaccine efficacy were particularly alarming. The Danish Prime minister informed the nation about these developments in two seperate press briefings. To halt the further spread of the mutated virus, it was announced that the seven municipalities with the highest prevalence of the mutated virus were to prepare for a strict lockdown with severe additional restrictions. Crucially, the selection of these seven municipalities was purely based on the prevalence of the mutated virus, while local infection levels did not play a role in the process.

By the end of October, all Danish municipalities were under a number of restrictions. These restrictions limited the size of public and private gatherings, mandated masks in public transport and retail stores and similar (for a detailed overview see Supplementary Information 1).

On the 9th of November, more far-reaching additional restrictions were added exclusively to the seven municipalities of concern. These additional restrictions included a ban on cross-municipal travel and public transport, work from home and home schooling and the closure of restaurants, bars and similar establishments (for a detailed overview see Supplementary Information 1).

## Results

**Figure 1:**
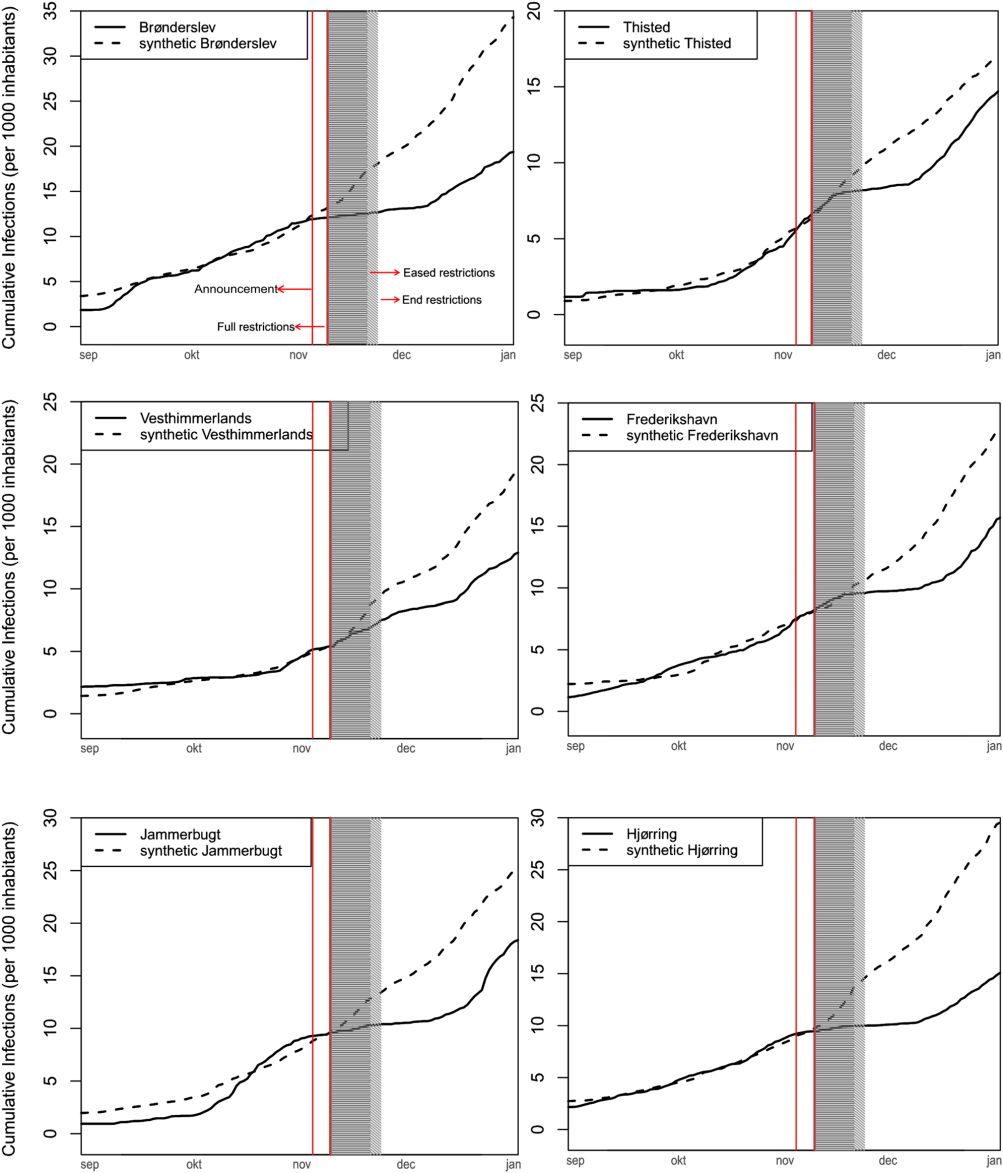
Actual vs. synthetic cumulative daily infections. For each of the six Northern Jutland Municipalities placed under additional restrictions, a synthetic counterfactual was created out of a weighted combination of the remaining 91 Danish municipalities. The aim of each counterfactual is to resemble the cumulative infection dynamic of the actual municipality in the pre-treatment period (September 1st to November 6th). The red line (9th of November) marks the first day on which restrictions are introduced, the grey area between November 9th to 20th is the period in which the full additional restrictions are in place, in the light-grey area between November 20th to 23rd they are being phased-out again.

Figure 1 presents the results of the counterfactual analysis for each of the six municipalities of interest. The synthetic control approach succeeded in creating synthetic municipalities with similar pre-treatment cumulative infection dynamics as each actual municipal counterpart. See “Materials and methods” for a detailed description of the data, the synthetic counterfactual estimation process and reasoning for excluding one of the seven municipalities restrictions were applied to.

All six municipalities exhibit a period where cumulative infections plateau, meaning no or hardly any additional new infections (defined as a prolonged (>3 days) period with less than 0.2 daily infections per 1000 population) were confirmed. In three cases [Brønderslev (31st Oct–7th Dec), Jammerbugt (31st Oct–10th Dec) and Hjørring (30th Oct–14th Dec)] this plateauing effect begins right before the beginning of the lockdown. In two cases [Thisted (16th Nov–9th of Dec) and Frederikshavn (14th Nov to 15th Dec)] a plateauing appears to begin around 10 days into the lockdown while in one case [Vesthimmerlands (3rd Dec–10th Dec)] it appears around 1 week after the end of the lockdown.

Defined as above, there is no post-treatment plateauing effect discernible in any of the synthetic municipalities (dashed lines), as would be expected given the absence of additional restrictions in the municipalities considered to create these synthetic counterfactual cases. On average, these plateauing periods lasted for about 16 days, after which infections again increased at similar paces compared to those of the counterfactuals.

By the end of the observation period on December 31st, 2020 the six municipalities reported on average 31% less total infections than their synthetic counterparts, with Hjørring Municipality reporting the highest reduction at 43% and Thisted Municipality reporting the lowest at 19%. These findings are in line with what has been reported in the literature in similar case studies around the world before vaccines became available^10,16–18^.

### Google mobility data

For a restriction to lead to decreased observed daily SARS-CoV-2 infection numbers, citizens must actually comply with the rules mandated by said restriction. Prior research from the US on the specific topic of adherence to COVID-19 restrictions has shown that individuals do reduce their daily mobility in response to both increased local infection numbers and official restrictions^19^. Further, research confirmed that reductions in citizens mobility does lead to decreases in infections^20,21^.

**Figure 2:**
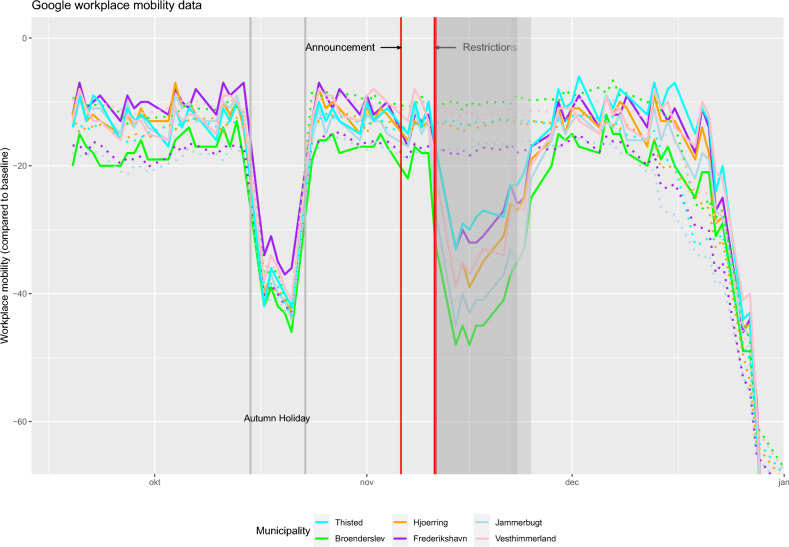
Google workplace mobility data. Relative mobility change of individuals physically spending time at their workplace, compared to a pre-COVID-19 baseline (January 2020). Two vertical grey lines mark the Danish autumn holiday (October 12–18), the first, thin, red line marks the announcement of restrictions by the Danish Prime Minister (November 4th) and the second vertical red line the beginning of the restrictions (November 9th). The grey zone indicates the period the restrictions were in place and then eased (November 20–23). For better visualization, weekends are omitted.

Accordingly, for the observed reductions in infections in the six municipalities of Northern Jutland to be a causal effect of the restrictions implemented, a reduction in citizens mobility following the restrictions would be expected. Figure 2 shows Google Mobility Data for the six municipalities from September 2020 through December. It is evident that the restrictions resulted in a substantial reduction in (workplace) mobility, i.e. time spent at the workplace. There is no reduction in mobility due to the restrictions in either of the synthetic municipalities. Before and after the period of additional restrictions, the actual and synthetic municipalities exhibited rather similar workplace mobility behaviour. The magnitude of workplace mobility reduction during the restrictions is between an additional 17% (Frederikshavn) to 37% (Brønderslev) compared to the January baseline. This additional mobility reduction is similar to the one observed in mid October during the Danish autumn holidays and can be considered substantial.

The adjustment to workplace mobility observed in the Google Mobility data begins on the first workday following the beginning of the restrictions. The mere announcement of the upcoming restrictions in the week prior does not result in any reductions to workplace mobility. The observed reductions to workplace mobility already begin to wean off later into the restriction period and normalize to the levels observed prior.

### SARS-CoV-2 test positivity rates

PCR tests can detect a SARS-CoV-2 infection after around 2–3 days, which, on average, is 2–3 days before the onset of symptoms^22^. Proper detection and official confirmation of infections is crucial for this study, as only officially reported positive infections are included in the SSI data used for Fig. 1.

**Figure 3:**
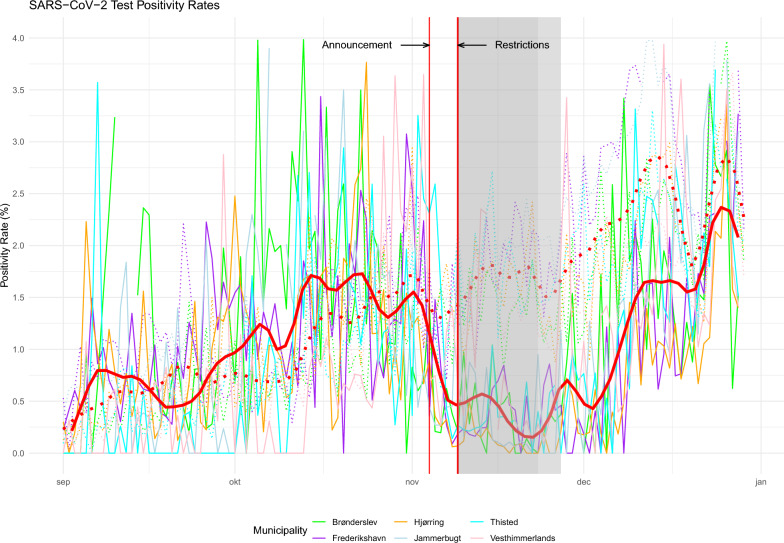
SARS-CoV-2 test positivity rates. Test positivity rates for all 6 municipalities (solid red moving line) and their corresponding synthetic counterfactual (dotted red moving line) using the synthetic weights derived for Fig. 1. The moving red lines represent the rolling average values of the actual (solid red) and the synthetic (dotted red) municipalities.

During the second wave of infections in Denmark, SARS-CoV-2 test positivity rates started a steady increase from the end of summer 2020 through December. As can be seen from Fig. 3 test positivity rates in the actual (solid red moving line) and synthetic municipalities (dotted red moving line) increased in parallel from September to November. Starting with the announcement of additional restrictions on November 4th test positivity rates in the actual six municipalities drop drastically. Test positivity rates can be influenced by two factors, a decrease or increase in actual infections and a decrease or increase in the number of tests being performed. In the announcement made on November 4th the Danish Prime Minister urged citizens in the concerned municipalities to get tested. On average in the six municipalities, in the period before the announcement, around 2500 tests were performed per day. This number increases drastically for the restriction period, averaging around 9500 tests per day instead. This can explain the sudden and rapid drop in positive SARS-CoV-2 tests for the six Northern Jutland municipalities occurring right after the announcement. Unlike workplace mobility, test positivity rates first assimilate to levels found in the synthetic counterparts towards the end of 2020 around Christmas.

## Robustness checks

### Pre-treatment fit

To ensure the results validity it is important to establish that relevant observable characteristics of the municipalities and their synthetic counterfactuals before the treatment are similar. Figure 1 allows for visual inspection of the pre-treatment similarities in cumulative infections per 1.000 inhabitants between the actual and the synthetic municipality. Furthermore, Table 2 allows for the comparison of additional observable characteristics between the actual and synthetic municipalities and also the overall average for all Danish municipalities (donor average). This is important as Danish municipalities are rather diverse, with for example population sizes varying between 3.000 (Ærø) to 600.000 (Copenhagen) and average female and male ages between 54 and 51 (Ærø) to 37 and 36 (Copenhagen).

The first additional observable characteristics reported in Table 2 are for specific infection numbers, firstly the average over the total pre-treatment period and next infection numbers for three individual days right before the implementation of further restrictions. Hereafter, similarity for the five control variables, education, average age for females and males, old age and young age dependency ratios, are reported. All 6 municipalities in question are characterised by a higher increase in cumulative infections towards the days leading up to the restrictions. Additionally, compared to the total donor average, they are characterised by lower levels of education and higher female and male average ages and old age dependency ratio, while young age dependency ratios are more varied. The synthetic counterfactual for each municipality is able to closely resemble the characteristics observed and also the overall pre treatment fit in Fig. 1 is able to closely capture the trends in the data. Based on this similarity in relevant observable characteristics, it is assumed that potentially relevant unobserved characteristics, such as for example population immunity levels, are also being (indirectly) controlled for by the method and the results are overall robust.

### Leave-one-out analysis

**Figure 4:**
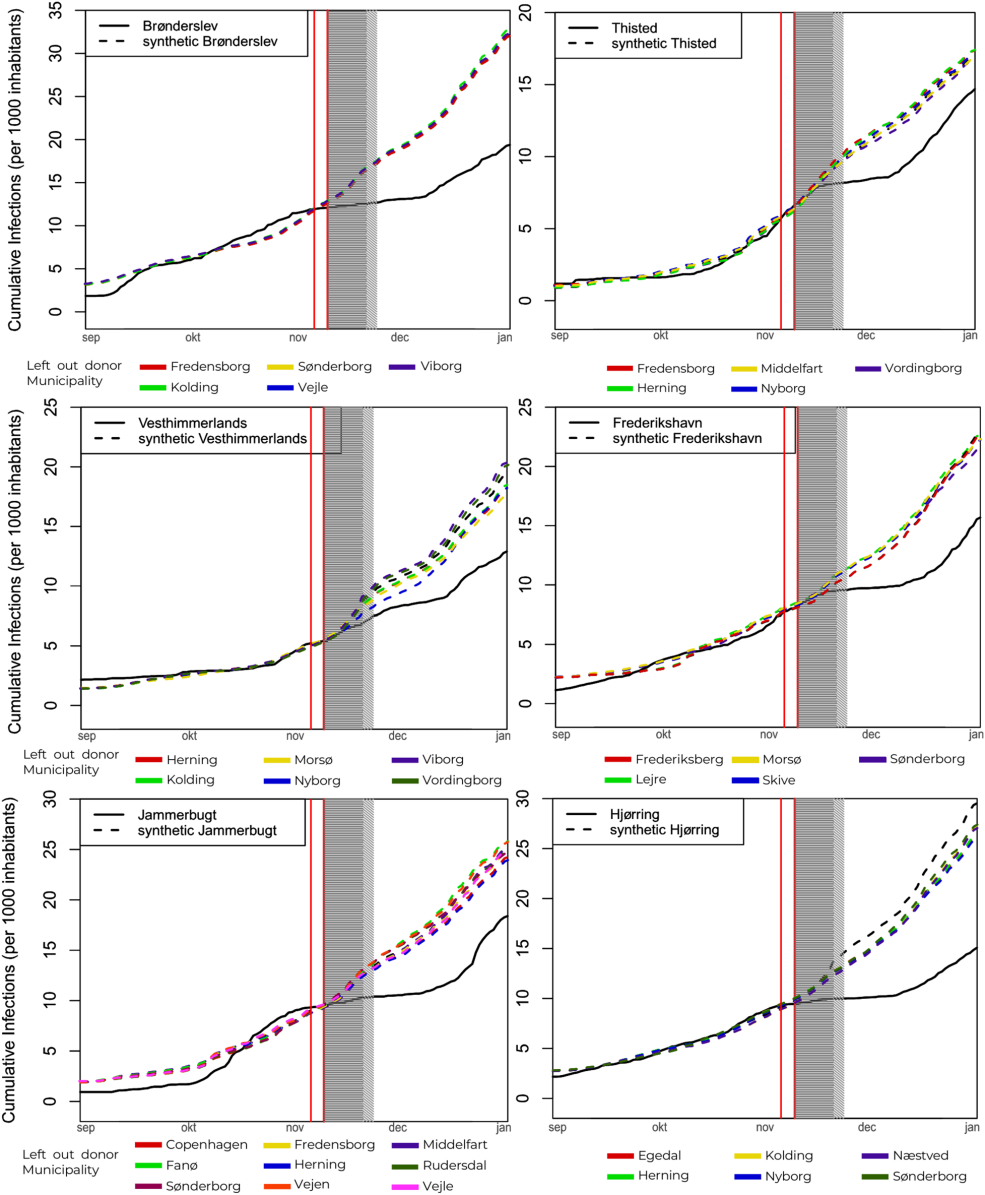
Leave-one-out tests. Rerunning the synthetic control analysis, each time dropping one of the weighted donor municipalities (see legend for which colour dotted line corresponds to which weighted municipality having been dropped from the donor pool) used in the original synthetic counterfactual (dotted black line).

Figure 4 presents the results for the leave-one-out analysis, a robustness check based on the main results presented in Fig. 1 and Table 1. For a synthetic municipality, one-by-one each contributing donor is excluded and the analysis is re-run without the excluded donor. Each of these leave-one-out analysis results is presented by a coloured line. Results can be regarded as robust when the coloured dotted lines reveal similar results as the original black dotted line does. As it is apparent in Fig. 4, all 6 results are robust to the exclusion of specific municipalities, meaning that the conclusions drawn from the analysis are not dependent on the inclusion of any one specific or set of municipalities to construct the synthetic counterfactual with^23^.

## Discussion

### Could the prevalence of different COVID-19 variants in Denmark explain the observed trends?

Genome sequencing of positive test samples was rather widely used in Denmark, so we know that there indeed were different variants prevalent at the time. An epidemiological study found that between 53% (in October) to 26% (in November) of cases in the Northern Jutland region were associated with the mink variant ’Cluster 5’^24^. However, crucially, the concern about the new variant was primarily its potential resistance to upcoming vaccines, while an increased transmissibility or more severe symptoms associated with the variant were not observed^25^. An earlier study also reported no evidence of increased transmissibility or more severe symptoms^26^. Additionally, if variation in the prevelant virus variant would have altered existing infection dynamics, this would have been the case during the pre-treatment period also, meaning that the counterfactuals created would have taken this into consideration and still chosen comparable municipalities with similar absolute infections. Also the parallel trend in test-positivity rates before the announcement of restrictions seen in Fig. 3 indicates that the prevalence of different virus variants had no direct effect on infection numbers.

#### Why do infection numbers in some cases begin to plateau already before the beginning of restrictions?

Indeed, as also highlighted previously^14^, in the municipalities of Brønderslev, Jammerbugt and Hjørring the plateauing effect of cumulative infection numbers can be observed to begin already a few days before the introduction of restrictions. However, as can be seen from Fig. 3, the first behavioural change in the form of increased testing activity begins not first at the day when restrictions are introduced (November 9th), but already when the upcoming restrictions are announced by the Danish prime minister (November 4th).

PCR-tests begin to be able to detect the SARS-CoV-2 virus three days after infection^27^. Therefore, it is plausible, especially given the drastic increase in testing activity, that the announcement of either the presence of a mutated virus or the upcoming restrictions already resulted in an observable decrease in infections few days prior to the restrictions being enforced. There are no indicators of changes to either test-positivity rates or workplace mobility prior to the announcement date.

Figure 1 illustrates that the synthetic counterfactuals for each municipality tend to extrapolate a rather linear trend. However, the accumulated daily infection data for each municipality showcases earlier described plateuing effect with no or very few new cases being registered during or right after the short lockdown. In the municipalities of Brønderslev, Jammerbugt and Hjørring this effect indeed appears to begin right around the announcement of upcoming restrictions. However, substantial variations between actual and counterfactual infection numbers can also in these three cases first be observed between one to two weeks after the announcement and accordingly are plausible effects of the announcement.

#### Are the synthetic counterfactual estimates and the results based on them credible?

Denmark is administratively divided into 98 municipalities of varying demographic and geographical sizes. While Copenhagen Municipality has a total population of over 600.000, some of the smallest Danish Municipalities have less than 5.000 inhabitants. This means that any comparison between them has to be respectful of these differences. The SCM approach ensures that the constructed counterfactuals are the best-fitting comparison based on similar pre-treatment trends.

As can be seen in Fig. 4 the synthetic counterfactual and the actual municipalities follow similar pre-treatment infection patterns and, as reported in Table 2, are also similar in relevant characteristics. The length of the pre-treatment period contains 67 days and could not meaningfully be expanded due to the near absence of infections during the Danish summer of 2020, but is similar in length or longer than in comparable studies^6,10,28^.

Additionally, further discussed in the “Materials and methods” section below, the use of infection numbers as outcome measure for the effectiveness of restrictions in this case is valid. This is due to the high and consistent testing rates found in Denmark at the time, combined with high-quality digital systems ensuring that the recorded infection data does not suffer from lag introduced by delayed processing^29^.

#### Can we be sure that the observed plateauing of infection numbers is due to the additional restrictions and not voluntary behavioural changes?

The question about the difference in effectiveness between voluntary or mandatory restrictions is not in itself the topic of this paper, but the question is still of relevance. The findings shown in Fig. 3 suggests that citizens were willing to voluntarily increase testing activity already prior to mandatory enforcement. On the other hand, the findings on citizens mobility, reported in Fig. 2, clearly indicate that adjustments to (workplace) mobility are the result of mandatory enforced restrictions that would not have occurred without formal enforcement as mandatory. These findings are in line with prior research on this topic that confirm a more drastic decrease of citizens mobility when restrictions are enforced as mandatory^21,30^.

## Conclusion

The analysis of the 2020 Danish mink lockdown of six municipalities conducted in this paper aimed at analysing the effectiveness of lockdown policies to reduce SARS-CoV-2 infections. This means that merely the policies ability to achieve the desired primary outcome (reduced infection numbers) is being considered, while considerations of any additional outcomes (for example, negative economic consequences or reduced mental health) are outside the scope of this study. Accordingly, this study can not evaluate whether implementing the additional restrictions in the six municipalities was a good idea or not, as these conclusions would require an overall analysis of the efficiency of the restrictions, considering both their effectiveness at achieving the desired outcome and weighing these against any potential additional outcomes, i.e. desirable and undesirable side-effects.

This study can also not be directly generalized to other contexts, as relevant observable and unobservable characteristics of the overall context and specific content of each case will vary.

Overall, the results of this study do show that the additional restrictions imposed were effective at reducing the municipalities daily infection numbers. By linking the restrictions to decreases in test-positivty rates as well as mobility the study is able to demonstrate that the observed reduction in infections were indeed linked to both announcement and formal enforcement of the restrictions. A precise evaluation of any restrictions effectiveness to directly achieve its desired outcome is a critical first step before being able to evaluate its overall efficiency at doing so. Here, the study showcases a rare example of a rather ideal quasi-experimental scenario to conduct such an analysis of COVID-19 restrictions effectiveness.

## Materials and methods

Using confirmed daily infection numbers per municipality provided by the Danish SSI and applying the synthetic control method (SCM) to derive credible counterfactual scenarios for the absence of an intervention, this study estimates the causal effect of a specific, stringent, yet short-term lockdown in selected municipalities in Denmark in late 2020.

The pre-treatment period considered in this study spans from September 1st to November 9th (a total of 70 days). September is typically considered as the beginning of “the second wave” of SARS-CoV-2 infections after a first wave in spring followed by a low infection period during summer. Restrictions were formally enforced starting November 9th, but were publicly announced some days prior on November 4th. Regarding COVID-19 restrictions, previous research has shown that desired behavioural adjustments by individuals, such as social distancing, increased testing etc., appear often to begin at the date of the announcement of a restriction rather than the date of its formal implementation^31^. Figures 2 and 3 showcase behavioural responses to restrictions, outlining daily workplace mobility and SARS-CoV-2 test positivity rates, respectively, and therefore both the date when restrictions are formally enforced and also the date when they were announced are marked in all Figures.

The additional restrictions applied to seven municipalities, one of these, namely Læsø municipality, is a small island with merely less than 2.000 inhabitants. Therefore, this municipality was excluded from the analysis and instead the remaining six municipalities were analysed individually. The remaining six municipalities have a population size between 12.000 to 58.0000 inhabitants each. Travel between any of these six municipalities was prohibited, so was mobility from any of the untreated 91 remaining municipalities into any of the ones targeted by the additional restrictions, allowing for each case to be analysed individually.

The main outcome analysed, numbers for officially confirmed infections, were strongly under-representing the actual infection burden during the first months of the pandemic, as testing availability necessary to confirm a positive infection, was lacking^32^. In the period from February to September 1st a mere total of 424 SARS-CoV-2 tests per 1.000 population were performed, the majority of which were taken in July and August. For the period from September 1st through December a total of 1376 tests per 1000 population were conducted, making Denmark the country with the highest per capita SARS-CoV-2 testing activity, second only to Luxembourg^11,12^. High free public testing activity is crucial, as only officially confirmed positive tests are included in the data analysed, yet even during the second wave in later 2020 testing availability varied greatly between countries and the high testing activity in Denmark was rather exceptional.

Synthetic Control, the method used to derive estimates of outcomes had additional restrictions not been implemented (dotted lines), is a method for causal inference similar to Difference-in-Difference (DiD), but instead of identifying a single untreated unit as counterfactual, synthetic control uses a weighted combination of untreated units to create a best-fitting counterfactual case. The method has been used to estimate the effectiveness of COVID-19 restrictions in numerous previous studies^6,7,30,33,34^. It is implemented using the Synth package in R^35^. In the framework of SCM, the outcome for the treated unit $i$ at time $t$, denoted as $Y_{it}^N$, is estimated by the linear combination of the outcomes from control units^36^. Mathematically, this relationship is represented as:

1 $$\begin{aligned} Y_{it}^N = \sum _{j=1}^J w_j Y_{jt}^N \end{aligned}$$

Here, $Y_{jt}^N$ indicates the observed outcomes for control unit $j$ at time $t$, and $w_j$ are the weights assigned to each control unit. The weights are chosen to be non-negative and their sum must equal one, ensuring that the synthetic control is a convex combination of the control units, in this case the 91 Danish municipalities not targeted by the stringent lockdown. For the weights chosen by the method for each synthetic counterfactual, see Table 1. The objective of SCM is to minimize the squared differences between the pre-treatment outcomes of the treated unit and the synthetic control. This is formulated as a minimization problem:

2 $$\begin{aligned} \min _{w} \sum _{t=1}^{T_0} \left( Y_{it}^N - \sum _{j=1}^J w_j Y_{jt}^N \right) ^2 \end{aligned}$$

where $T_0$ is the number of pre-treatment periods. This optimization ensures that the synthetic control provides the best possible approximation of the treated unit’s trajectory in the absence of the intervention. The methods successfullness in doing so can be visually observed as the similarity in the pre-treatment period between actual and synthetic trends of Fig. 1.

Next to trying to recreate similar cumulative infection numbers in the pre-treatment period, the method also tries to ensure that synthetic municipalities should resemble each municipality on a number of additional control variables. These additional controls include education levels, average female age, average male age, old age dependency ratio and young age dependency ratio. As can be seen from Table 2, the model succeeded at recreating synthetic municipalities similar in terms of these control variables.

**Table 1:** Weighted combinations of non-treated Danish municipalities to create each synthetic municipality.

	Synthetic Brønderslev
Viborg	53.1 %
Vejle	22.6 %
Sønderborg	10.3 %
Kolding	7.7 %
Fredensborg	5.2 %

	Synthetic Thisted
Herning	44.6 %
Middelfart	28.6 %
Vordingborg	15.1 %
Fredensborg	9.2 %
Nyborg	1.5 %

	Synthetic Vesthimmerlands
Nyborg	32.0 %
Viborg	29.8 %
Herning	12.8 %
Morsø	9.9 %
Kolding	2.8 %
Vordingborg	2.5 %

	Synthetic Frederikshavn
Sønderborg	33.7%
Frederiksberg	31.2%
Morsø	22.8%
Lolland	9.6%
Skive	2.1%

	Synthetic Jammerbugt
Copenhagen	29.3%
Sønderborg	25.8%
Middelfart	9.9%
Fanø	9.5%
Fredensborg	7.5%
Vejle	6.7%
Vejen	1.7%
Rudersdal	1.6%
Herning	1.6%

	Synthetic Hjørring
Nyborg	34.6%
Sønderborg	24.0%
Egedal	19.3%
Herning	10.6%
Næstved	6.4%
Kolding	1.2%

**Table 2:** Balance on baseline characteristics.

	Brønderslev	Synthetic	Donor average
Infections (average)	9.3	8.7	6.3
Infections (7.11)	12.1	12.4	8.8
Infections (6.11)	12.0	12.3	8.7
Infections (5.11)	12.0	12.0	8.6
Education	0.04	0.05	0.07
Avg. age females	44.3	44.1	44.7
Avg. age males	42.7	42.6	42.8
Old age depend.	0.37	0.37	0.37
Young age depend.	0.29	0.29	0.27

	Thisted	Synthetic	Donor average
Infections (average)	3.3	3.5	6.3
Infections (7.11)	6.4	6.2	8.8
Infections (6.11)	6.3	6.0	8.7
Infections (5.11)	6.0	5.9	8.6
Education	0.03	0.03	0.07
Avg. age females	45.5	45.5	44.7
Avg. age males	43.9	44.0	42.8
Old age depend.	0.40	0.40	0.37
Young age depend.	0.27	0.27	0.27

	Vesthimmerlands	Synthetic	Donor average
Infections (average)	3.6	3.6	6.3
Infections (7.11)	5.4	5.3	8.8
Infections (6.11)	5.3	5.2	8.7
Infections (5.11)	5.2	5.1	8.6
Education	0.03	0.03	0.07
Avg. age females	45.7	45.7	44.7
Avg. age males	44.1	44.1	42.8
Old age depend.	0.40	0.40	0.37
Young age depend.	0.27	0.27	0.27

	Frederikshavn	Synthetic	Donor average
Infections (average)	5.4	5.5	6.3
Infections (7.11)	8.0	8.0	8.8
Infections (6.11)	8.0	7.9	8.7
Infections (5.11)	7.8	7.8	8.6
Education	0.03	0.03	0.07
Avg. age females	48.1	47.9	44.7
Avg. age males	45.9	46.0	42.8
Old age depend.	0.45	0.46	0.37
Young age depend.	0.24	0.24	0.27

	Jammerbugt	Synthetic	Donor average
Infections (average)	5.9	6.2	6.3
Infections (7.11)	9.4	9.4	8.8
Infections (6.11)	9.4	9.4	8.7
Infections (5.11)	9.4	9.2	8.6
Education	0.04	0.04	0.07
Avg. age females	45.6	45.6	44.7
Avg. age males	44.0	44.0	42.8
Old age depend.	0.41	0.41	0.37
Young age depend.	0.28	0.28	0.27

	Hjørring	Synthetic	Donor average
Infections (average)	7.0	6.8	6.3
Infections (7.11)	9.4	9.6	8.8
Infections (6.11)	9.4	9.4	8.7
Infections (5.11)	9.4	9.3	8.6
Education	0.3	0.03	0.07
Avg. age females	45.8	45.7	44.7
Avg. age males	43.8	43.9	42.8
Old age depend.	0.39	0.40	0.37
Young age depend.	0.26	0.26	0.27

See Table 1 for the composition of each synthetic municipality. Donor average refers to the average value found for all untreated 91 Danish municipalities considered for constructing the synthetics.

### Supplementary Information

Supplementary Information 1.

Supplementary Information 2.

Supplementary Information 3.

Supplementary Information 4.

Supplementary Information 5.

## Data Availability

The datasets analysed in the current study are publicly available at either the Danish Statens Serums Institue (SSI), which can be accessed here: https://files.ssi.dk/covid19/overvagning/data/overvaagningsdata-covid19-31122021-kzmk, or Google’s regional Community Mobility Reports, available here: https://www.gstatic.com/covid19/mobility/Region_Mobility_Report_CSVs.zip.
