# Supplementary material for: Short, stringent lockdowns halted SARS-CoV-2 transmissions in Danish municipalities
Source: Sci Rep. 2024 Aug 12;14:18712. doi: 10.1038/s41598-024-68929-z (PMC11319722; doi:10.1038/s41598-024-68929-z)
Supplement: Supplementary file 1 — Supplementary Information 1. [file 41598_2024_68929_MOESM1_ESM.pdf]

Machine Translated by Google

[corona | covid-19]

# Restrictions

from 26 October 2020

(Mask rules from 29 October)

**Assemblies**
Ceiling on gatherings of 10 people. This also applies to private parties held outside private homes and gardens. There are exceptions, see other points.

**Private events and social contacts**
Recommendation of max. 10 people in a private home and max. 10 social contacts. Limit social events.

**Collective transport**
Requirement for mask or visor.
Avoid rush hour. Requirement for a seat ticket on long-distance trains.

**The retail trade**
**(Grocery stores, department stores, department stores, bazaars, arcades, etc.)**
Requirement for mask or visor. Prohibition on the sale of alcohol after 10 p.m. Restrictions, e.g. distance and number. Requirements for visible supervisory staff in stores of over 2,000 m2.

**The health and care sector**
Requirements for masks or visors in the health and elderly areas as well as certain parts of the social area.

**Church and religious community**
Max. 500 present at the same time (however depending on the size of the room). Max. 50 people at outdoor funerals.

**Workplaces**
Homework to the extent that it is possible and appropriate in relation to the work. Recommendation to cancel social events.

**Day care and primary schools**
Staff must wear a visor.

**Youth education, adult education, further education, etc.**
Requirement for partial use of mask or visor.

**Sports and association activities**
Ban on gatherings of 50 people for sports activities for children and young people. Other sporting activities are subject to a ban on gatherings of 10 people.

**Restaurants, cafes, etc.**
Must close at 10 p.m. Requirement for mask or visor indoors - but not when sitting down. Companies must be of max. 10 people.

**Bars, pubs, etc.**
Must close at 10 p.m. Requirement to use a mask or visor - but not when sitting down.

**Venues, conferences, etc. with seated audience**
Max. 500 people if seated and facing a stage, cinema screen, track or similar.

**Discotheques, nightclubs, etc.**
Closed. In addition, a ban on the consumption of alcohol on buses – including party buses.

**Indoor cultural institutions and sports facilities**
Requirement for mask or visor. Restrictions, e.g. distance and number.

**Professional sports**
Max. 500 people if seated and facing a stage, cinema screen, track or similar.

Hotline: +45 7020 0233
**coronasmitte.dk**

SUNDHEDS-
OG ÆLDREMINISTERIET

POLITI
