# Supplementary material for: Short, stringent lockdowns halted SARS-CoV-2 transmissions in Danish municipalities
Source: Sci Rep. 2024 Aug 12;14:18712. doi: 10.1038/s41598-024-68929-z (PMC11319722; doi:10.1038/s41598-024-68929-z)
Supplement: Supplementary file 2 — Supplementary Information 2. [file 41598_2024_68929_MOESM2_ESM.pdf]

[corona | covid-19]

New restrictions and recommendations in seven North Jutland municipalities

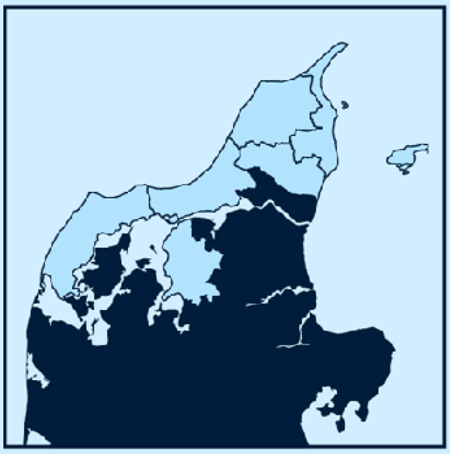

Valid from 9 November 2020, except restrictions for restaurants, cafes, bars, pubs etc., which apply from 7 November 2020

The seven municipalities are Hjørring, Frederikshavn, Læsø, Brønderslev, Jammerbugt, Thisted and Vesthimmerland.

Assemblies
Ceiling on gatherings of 10 people. This also applies to private parties held outside private homes and gardens.

Mobility
Residents of the seven municipalities are strongly encouraged not to cross municipal borders, except for essential and urgent reasons. Everyone else is strongly encouraged not to visit the mentioned municipalities.

Massive testing effort
Everyone residing in the mentioned municipalities is encouraged to be tested. There will be local announcements that you should wait for.

Workplaces
Public and private workplaces are strongly encouraged to send home all employees who do not carry out critical functions. Public and private workplaces outside the municipalities in question are encouraged to send all employees residing in the municipalities in question who do not carry out critical functions home.

Day care, primary school and youth education, etc.
Grades 5-8 are sent home for distance learning. Other classes can have attendance, but with a heightened focus on keeping distance, hygiene etc. Attendance is reduced to 50% at youth and adult education etc.

Higher education etc.
Students with residence in one of the mentioned municipalities transfer to digital teaching and exams without attendance. It also applies for students in higher education educations in Aalborg, who reside in the municipalities concerned.

Cultural institutions
All cultural institutions and premises where exercise is carried out cultural activities, are closed to public access. That applies to both indoor and outdoor areas.

Restaurants, cafes, etc.
Must close for serving to consumption at the point of sale (however incl possibility of take-away).

Bars, pubs, etc.
Must close for serving to consumption at the point of sale (however incl possibility of take-away).

Collective transport
Public passenger traffic is closed with e.g bus, train and ferry in and out and across them mentioned municipalities, but not for driving with school bus and the like.

Sports and leisure facilities
Indoor premises where exercise is carried out sports, association and leisure activities, be closed. In outdoor activities, max 10 people participate.

Professional sports
The exception for events of up to to 500 seated guests are removed. Thus is closed to spectators.

See further details and possible exceptions at coronasmitte.dk

Hotline: +45 7020 0233
coronasmitte.dk

HEALTH
AND THE MINISTRY OF THE ELDERLY

POLICE
